# Analectic

**Published:** 1836

**Authors:** 


### MISCELLANEOUS INTELLIGENCE.

### ANALECTIC, ANALYTICAL, AND ORIGINAL.

### I.—ANALECTIC.

*Ad Sylvas Nuncius.*

1. *Treatment of Paralysis from disease of the Arterial System.* By William Stokes, M. D.—With respect to the treatment of paralysis, if the patient be young, and the disease recognized at an early stage, it is possible that you may be able to arrest it by free local depletion, and other antiphlogistic means. In the case which was under treatment in the Meath Hospital, the symptoms had lasted for a considerable time before the disease exhibited any remarkable violence. The man was admitted on the 7th of February, and at this time the disease had been five weeks in existence, having begun at the lower part of the limb, and extended gradually upwards, until it envolved the whole leg and thigh. Yet it is very probable that this patient might have been saved, if proper means had been taken to arrest the inflammation of the vessels at an early period. Baron Dupuytren has published a case, in which it appeared that this disease was setting in, but was checked at once by bold antiphlogistic treatment directed to the affected limb.—*London Med. and Surg. Journ. Sept. 6th,* 1834.—*Amer. Jour. of the Med. Sciences, Nov.* 1835.

2. *On the employment of Blood-letting in Scarlet Fever.*—The *Edinburg Medical and Surgical Journal* for July last, contains an interesting communication from Mr. A. Dewar, of Dunfermline, on this subject. A dissection made of a child which had died of scarlet fever on the sixth day of the disease, and which exhibited unequivocal evidences of inflammation of the mucous membrane of the fauces, pharynx, trachea, bronchi, stomach, parts of small intestines, and brain, made early in life a strong impression upon Mr. D. and led him to doubt whether the remedies usually employed were sufficient to conquer the inflammatory action, which, he regards as the cause of the morbid phenomena of the disease. The many mortifying failures which he experienced in his own practice and witnessed in that of others, led him many years ago to resort to general bleeding in the treatment of the disease.

"During the last twenty years," says Mr. D. "I have witnessed four epidemics of scarlet fever, and for two years past it has prevailed, and still continues to prevail, to a great extent in this town and neighborhood. Since the 1st of July, 1833, I have attended 183 persons laboring under this disease in its acute stage, *i. e.*, with the eruption *still present* upon the body, and out of that number it has been my good fortune to lose only *two*. Nor must this small degree of mortality be altogether ascribed to comparative mildness in the character of the disease. As a proof that the present epidemic has not been a slight one, I may mention, that, in this town and its vicinity, upwards of 150 persons have died of scarlatina within the last two years.

"The gratifying success now mentioned, with which the cases occurring in my practice have been attended, is to be attributed to the early and efficient employment of general blood-letting. In every case in which the remedy was properly used, I have invariably found the symptoms greatly mitigated, and in many the disease wholly and suddenly subdued. To accomplish this purpose, however, a scanty or long-deferred blood-letting will not suffice. It must be practised, as in other cases of acute inflammation, so as to produce a marked impression on the circulation, while the quantity drawn must be so considerable as to make it probable that the impression will be permanent. When, therefore, I had an opportunity of seeing the case from the commencement, I preferred drawing blood when the eruption had decidedly appeared upon the breast, but had not spread over the rest of the body. I selected this time chiefly to prevent all ambiguity respecting the nature of the disease. I likewise preferred the evening for the purpose, as I generally found, that, if the bleeding succeeded in breaking the force of the fever during the evening exacerbation, the disease was more certainly subdued than if the blood was drawn during the morning remission. On all occasions I have found it necessary to bleed, whatever was the age of the patient, to complete relaxation. The bleeding when thus practised is immediately followed by diminution of the heat of the body, of the force and frequency of the pulse, and of the head-ache and sore throat; and the eruption entirely disappears, and in many cases scarcely again becomes visible. While the blood yet flowed, many patients have expressed in strong terms the relief they enjoyed. I myself passed through the disease in November, 1833, and can speak from personal experience of the soothing influence which the remedy exerts over the feelings of the sick. None can know but those who have felt it, the sudden and delicious transition which I experienced on being bled, from burning heat, general restlessness, and confused thoughts, to perfect quietude and self-possession."

Mr. D. was for some time in the practice of setting the patient erect, and allowing the blood to flow until paleness, faintness or vomiting was produced. This he has more recently found not to be sufficient. The mere relaxation, especially, if it has been produced by the loss of a small quantity of blood, seems to produce only a very temporary interruption to the progress of the disease; from this state the patient soon rallies, and the fever resumes its force. Of late Mr. D. has bled all persons who had passed the period of mere infancy in the horizontal posture, and when he has obtained what he considers a proper quantity of blood, he raised them into the erect posture till relaxation took place. In this way he thinks he has avoided the necessity of repeating the bleeding.

Although the commencement of the eruption is without doubt, the time at which blood-letting can be practised with the greatest success, yet, while the pulse continues strong, and there is reason to believe that effusion has not taken place in the head, Mr. D. has not hesitated to bleed, and has never had reason to regret it, indeed he says he has succeeded on several occasions, where he is convinced no other remedy would have availed to save the life of the patient.

"Ulceration of the throat," observes Mr. D. "is one of the most severe and painful symptoms that accompany this disease. It is the consequence of the inflammatory action which prevails throughout the whole of the mucous membrane. Though it is probable the ulcerations are more frequent in the throat than in any other part of the mucous membrane, yet, they are, without doubt, often to be found in the bronchial membrane and intestinal canal; and I am convinced, that, if dissection were carefully performed, they would be found to prevail more generally and extensively than has hitherto been believed. No treatment can be more unsatisfactory than the leeches, the gargles, and the liniments, which are in common use for the cure of this untoward symptom. In truth, the treatment of the sore throat proceeds upon a defective knowledge of the pathology of the disease. The depletion is directed to the throat, as if it was the only part of the system laboring under inflammatory excitement, while in reality it is only one link in the chain of general inflammation in that particular structure. If this statement require confirmation, it is obtained in the fact, that, since I have employed general bleeding in the manner described, I have not had occasion in any of the 183 cases I have mentioned, with a single exception, to apply a leech for the cure of the sore throat, although I have seen several die of ulceration in the throat, who had been treated in the usual way. In every instance the pain in the throat was relieved by the bleeding, and readily and speedily yielded to the most ordinary means, and indeed, in a great majority of the cases, required no special treatment at all.

"I have invariably bled the patient until the eruption *disappeared*, and in a considerable proportion of the cases it did not return, and even when it did return, it was much less diffused and less fiery in appearance. The scholastic and imaginary terrors, lest the eruption be interfered with, I have long disregarded. I look upon the eruption as the evidence of the inflammatory state, and the fever as symptomatic of the inflammation; and I find that the eruption and the fever for a certain period advance or subside together. It is true that the fever often continues after the eruption has ceased, but in these cases it is prolonged by organic lesions produced by the inflammatory action in some internal structure. Out of the 183 cases, 147 were bled, and in all the eruption was essentially diminished, in many finally removed; yet not in a solitary case did any unpleasant consequence follow. The recoveries were uniformly rapid.

"Some speculatist will probably say, that, as the morbid matter is prevented by the bleeding from 'coming out,' the person will not be protected from a recurrence of the disease. Time alone can decide on the validity of this objection. I can only say in answer to it, that I have now known many persons who were thus treated, exposed, again and again, to the contagion, without being affected by it.

"It is gratifying to add, that I have not had even the approximation to a dropsical symptom. I have seen during the last two years fifteen cases of dropsy after *scarlatina;* but they all occurred in persons who had either received no medical treatment, or in whom the treatment had been conducted on different principles from that which I have been recommending. In none of these 183 cases has there been the slightest tendency to dropsical effusion.

"The two cases which I saw *during the eruption*, and in the treatment of which I proved unsuccessful, were instances of congestive fever. The onset of the fever was severe, and the reaction imperfect. The eruption appeared, but was pale, and fluctuated; and the face, hands, and feet, continued cold throughout the disease. The one died on the 4th day, the other passed on to deep typhus, and ultimately sunk apparently from exhaustion.

"The discredit into which blood-letting has fallen in the treatment of scarlet fever, has probably arisen from the want of due attention to the circumstances in which it has been prescribed. It is a remedy of great efficacy for good or for evil. If used at random, and without a careful discrimination of the circumstances of every individual case, it will, assuredly, justify the reprobation which Dr. Currie has thrown upon it; and may prove 'a fatal practice.' If used early, and in insufficient quantity, it will only diminish the strength of the patient, without lessening the force of the fever; and if delayed too long, it will accelerate the effusion into the head, to which the disease is fast hastening. If, on the other hand, it is practised at the proper time, and in sufficient quantity, it will prove a means of cure, safe and successful, far beyond any other with which I am acquainted.

"It will be objected, I dare say, that depletion, more especially ample depletion, will be unsuitable to some epidemics. There are certain conditions of the atmosphere, or rather certain unknown causes, under the influence of which, scarlet fever, (and indeed any other fever,) may assume an unusually malignant character. By this I understand that these causes, whatever they are, so modify the contagion, or the health of the individual, that the early stage of the disease is of unusually short duration, and the worst, or typhoid stage, is speedily developed. This I am willing to admit; but I believe it to be equally true, that the essential character of scarlet fever, I mean tbe inflammatory state is still present, and can be most successfully combated by its only appropriate treatment,—blood-letting; although certainly, greater promptitude and discretion are necessary in its application. The typhoid state is not the disease, but its consequence, and can only be prevented by removing the previous excitement. The free but cautious and seasonable depletion, which has robbed typhus itself of many of its terrors, would, I doubt not, be more successfully directed to the cure of malignant scarlatina, than all our other remedies put together."

Mr. D. has related several cases illustrative of the prompt benefit resulting from his method of treatment, two or three of which we subjoin.

"Miss G., aged thirteen, was seized on the evening of the 30th of March, 1834, with languor, pain in the back, and sore throat. During the night she was very hot, extremely restless, and the morning of the 31st in some degree incoherent. I saw her in the afternoon. Her face was flushed, her eyes red, she tossed in bed, was drowsy, and it required an effort to rouse her to consciousness. Her neck was stiff; her throat swelled, very red and painful; skin intensely hot; pulse 125, and strong; her neck, chest, and arms, were covered with the eruption of scarlatina, to the contagion of which she had been exposed. She was raised in bed, and bled to about a pound, when she became faint and the eruption entirely disappeared. She expressed great and instant relief from the bleeding. Two hours after she sat up in bed and took tea. About nine at night a pailful of milk-warm water was poured over her body, after which she slept well. Next morning her pulse was 90, the pain of the throat greatly relieved, and her general feelings very comfortable; the eruption scarcely perceptible. A tea-spoonful of Epsom salts acted gently on her bowels. At night the tepid effusion was repeated. Next day she was so well as to be occasionally out of bed. Took gruel and tea with toast for food. No other remedy was used. On the fifth day from the attack she was able to leave her apartment."

"In the autumn of 1833, I was requested to visit a young woman, aged fifteen, on the fifth day of her confinement to bed, and the third from the appearance of the eruption. She lay prostrate in bed, was drowsy, and could with difficulty be roused to even imperfect consciousness. After a few moments of comparative stillness, she tossed from side to side in bed, and moaned constantly. The restlessness and moaning, with scarcely any cessation, had continued during the preceding twenty-four hours. Her eyes were red, the vessels of the conjunctiva being minutely injected with blood. Her skin was intensely hot, and impressed the fingers with a very peculiar pungent sensation. The distinct eruption had disappeared, but there remained on various parts of the body, particularly the arms and legs, large patches about half the size of the hand, of a dark red colour, resembling that of venous blood. The tongue was red, and universally excoriated. She complained when the epigastrium was pressed upon. The arterial pulsation in every part of the body was forcible and frequent. The danger was most imminent. Without delay, but not without hesitation, I drew blood from the arm. When about ten ounces had flowed she became faint. This quantity I regarded as insufficient to break the force of the disease. I therefore took the pillows from beneath her head, and put my finger on the wound till she rallied, and then withdrew other ten ounces of blood. After the bleeding she slept, and seemed more placid. In five hours the strength of the pulse and heat of the skin had returned, when she was again bled to a pound. During the night she was quiet, perfectly coherent, and slept occasionally. In the morning she was so collected as to give a distinct account of her sensations. She complained of pain, and a sense of knocking within the head; her sight was perfect, and the color of the eye was natural, but the expression wild. The heat of the surface was still very great; but the red patches had entirely disappeared. The pulse was strong and frequent; and the tongue raw. I again bled her to about twelve ounces, and gave her after a short interval, a small dose of Epsom salts in large dilution. From this time the fever did not return with any violence. The only other remedies were tepid washing for several nights, and small doses of sulphate of magnesia to regulate the bowels. She recovered rapidly.

"On the 20th of January, 1835, a boy, five years of age, was confined to bed with a hot skin, sore throat, great thirst, strong pulse, restlessness. He was covered with the eruption of scarlet fever. He was bled to faintness. In the evening I found him running about the room. He was washed at bed time, and took a small dose of castor oil. No other remedy was used. Next day he was in the parlor."—*Ibid.*

3. *On the employment of Sulphate of Copper as a remedy in Croup.*—The praises lavished on the use of sulphate of copper against croup by Dr. Hoffman of Darmstadt, in 1821, in Hufeland's Journal, and reiterated in 1826 in the Journal of Harless, determined Dr. Droste to subject it to trial; and this he has done for the space of seven years, apparently with great success; since during that time he has not lost a single patient,—a very extraordinary circumstance, even allowing that several of the cases were not severe. He states, that he has himself been surprised at the celerity with which the alarming symptoms were, as it were, extinguished after the administration of this salt. He has not recognised in calomel the efficacy ascribed to it by several distinguished practitioners. The first objection to its employment is that it acts too slowly; the second, its action upon the lymphatic system, when administered in large doses or repeatedly, or for a long time. In the latter case he believes that it renders the fibrine of the blood less plastic and more watery, and the children become cachectic and dropsical.

As croup owes its existence to an inflammatory irritation of the mucous membrane of the air-passages, which induces a profuse secretion of lymph, or mucus susceptible of being converted into false membrane, moulded on the laryngeal and tracheal canal, the first indication is to rescue the patient from the imminent danger of suffocation by clearing the air-passages from the mucus with which they are filled, and the false membrane, by which they are lined. This is not effected by means of calomel, unless at the same time be employed its ordinary adjuvants, as blood-lettings, emetics, rubefacients, vesications, and revellents; while the sulphate of copper in a large majority of cases is alone successful in effecting this object.

A full dose of the salt, that is, one, two, or three grains, produces immediately, or at the end of some minutes, violent vomiting, which forcibly detaches the false membranes; and, however insufficient the dose may have been, it is unnecessary to administer a second. Other emetics, on the contrary, when given in a large dose, often occasion *hyperemesis*, their action continues long, they induce diarrhœal symptoms, always useless, often dangerous, and in no case do they act according to the representation of Droste, so efficiently as the sulphate of copper. In a small dose, blue vitriol maintains a slight uneasiness, which terminates in profuse transpiration, or vomiting, which finally detaches the rest of the plastic exudation, which is heard wavering in the windpipe and *bronchi.* The voice then becomes clear, and respiration is more easy.

Weinhold admits that copper in a small dose, that is, in a dose too weak to excite vomiting, exercises no deleterious influence on the organism, but that it becomes dangerous when vomiting follows its administration. The contrary proposition appears to be the true one; for in a small dose it is absorbed, and gradually induces danger, especially if the organism be not accustomed to resist the injurious influence of external agents, unless, it is added, the remedy be administered in the homœopathic dose, in which case, though it does no benefit, because in the infant *belief is inert.*

Dr. Droste does not allow with Dr. Hoffmann, that the employment of the sulphate of copper supersedes the use of other remedies, as local bleeding by means of leeches, if the symptoms demand such remedies. In point of fact, in his cases, he orders not only local bleeding, but the application of blisters. He employs also powder of *digitalis*, or tartrate of antimony as antiphlogistics, subcarbonate of ammonia as an expectorant, purgative glysters, and the *potio Riverii* in some cases, and *spiritus Mindereri* in others.

The method of treatment consists in giving at the commencement one grain of blue vitriol to an infant under two years, two grains to a child of from two years to four, and three grains to a child between that period and eleven; a dose which is followed by almost immediate vomiting. If by this method the symptoms are not entirely subdued, sulphate of copper is then given in doses of one-fourth or one-half of a grain every two hours, according to its effects and the abatement of the disease. Of these small and subsequent doses, it appears from the cases to be rarely requisite to give more than three or four.

Such are the reported effects of this remedy and method of treatment, as represented by Dr. Droste. It is much to be desired that the author had been a little more detailed and particular as to the exact characters of the cases, as to whether he had really never failed in curing croup by this remedy, as to the subsequent course of the cases, and whether any effects were left by the action of the blue vitriol upon the stomach. We must say, that a detail of the effects of a remedy in which there had been some unsuccessful cases would have been more calculated to produce a favorable impression than one in which there are no instances of failure. Cases of invariable success inevitably lead to the conclusion, that many of them must have been very slight, and that the remedial agent employed had little to do with the result. They prove, in fact, too much.—*Edin. Med. and Surg. Journ.* *July*, 1835, *from Heidelberg Clin. Annals, Vol. X. No.* 2, 1834.

4. *On the Changes produced in the Composition of the Blood by repeated bleedings.* By THOMAS ANDREWS, Esq.—The object of the following experiments is to determine with precision the changes which are produced in the composition of the blood by repeated abstractions of large quantities of it from the general circulation. In the human subject, opportunities seldom occur of procuring proper specimens for examination, although the operation of venesection is so frequently performed; as in those cases where it requires to be repeated at short intervals the blood is generally in a morbid state. Instead of waiting for such casual occasions, I directed my attention to those animals in which the composition of the blood is nearly the same as in man, conceiving that similar results would in either case be produced. I selected the blood of calves for the purpose of experiment; and as it is the practice of butchers in this country to bleed these animals several times before they are slaughtered, I availed myself of this circumstance to procure suitable portions of blood. The animal is bled from a large orifice in the jugular vein, till symptoms of syncope appear, and the operation is in general repeated at intervals of twenty-four hours. It is once fed between each operation upon a mixture of meal and water, but this is often omitted before the last bleeding.

The appearance of the blood becomes greatly altered by the successive abstractions; the crassamentum is at first very large, and a portion of the red globules are unattached to it, but it progressively diminishes in bulk, while its consistency increases, till upon the fourth bleeding it appears a small contracted ball immersed in a large quantity of serum, adhering to the stopper of the vessel in which it is contained, and presenting on its external surface an exact cast of the interior of the vessel.

The following analyses were performed by the same method that I formerly employed in a set of experiments on the blood of cholera patients, which were published in the Philosophical Magazine for September, 1832. They are nearly all a mean of two separate analyses, which seldom differed from each other more than 0·5 per cent.

A calf was bled four times; between the first and second bleedings a week elapsed, but the rest took place at intervals of twenty-four hours, and the animal was fed between each operation. The composition of the serum and blood at each bleeding is exhibited in the following tables:

SERUM.

| | FIRST. | SECOND. | THIRD. | FOURTH. |
|---|---|---|---|---|
| Water - - - - - - | 92·19 | 93·96 | 93·81 | 94·18 |
| Albumen and Salts - - | 7·82 | 6·04 | 6·19 | 5·82 |
| | 100·00 | 100·00 | 100·00 | 100·00 |

BLOOD.

| | FIRST. | SECOND. | THIRD. | FOURTH. |
|---|---|---|---|---|
| Water - - - - - - | 81·36 | 85·49 | 87·41 | 89·25 |
| Albumen and Salts - - | 6·89 | 5·50 | 5·77 | 5·52 |
| Red Globules and Fibrin | 11·75 | 9·01 | 6·82 | 5·23 |
| | 100·00 | 100·00 | 100·00 | 100·00 |

The serum had at the third bleeding a specific gravity of 1·020, and at the fourth, of 1·017. At the third bleeding, the specific gravity of the blood itself, was 1·031.

The next calf whose blood was examined was nine weeks old. I did not procure any blood from the first bleeding. The third bleeding was twenty-four hours after the second, and during that period the animal was once fed; twelve hours afterwards it was bled a fourth time, but it received no more food:

SERUM.

| | SECOND. | THIRD. | FOURTH. |
|---|---|---|---|
| Water - - - - - - - - - - - - | 93·32 | 94·39 | 94·59 |
| Albumen and Salts - - - - - - | 6.68 | 5·61 | 5·41 |
| | 100·00 | 100·00 | 100·00 |

BLOOD.

| | SECOND. | THIRD. | FOURTH. |
|---|---|---|---|
| Water - - - - - - - - - - - - | 82·05 | 89·14 | 88·92 |
| Albumen and Salts - - - - - - | 5·85 | 5·29 | 5·06 |
| Red Globules and Fibrin - - - | 12·10 | 5·57 | 6·04 |
| | 100·00 | 100·00 | 100·00 |

The albumen and salts, it is evident, decrease at each bleeding ; the diminution is, however, very variable, and even after the fourth time does not amount to one per cent. and a half. In the globules, the same diminution takes place, but to such a degree that they are at least reduced to less than one-half their original quantity. To this principle a remarkable exception occurs in the composition of the blood taken at the last bleeding of the second calf, where the globules are slightly increased above the preceding analysis ; but it will be observed that the animal received no food during the intervening period, from which the blood might obtain a fresh supply of serum, while the tendency of the different excretions of the animal was to drain from the circulating mass its aqueous part, and thus to increase the apparent quantity of the globules. This explanation is confirmed by the following analysis.

A calf, three weeks old, was bled twice before it was killed ; twelve hours elapsed between the two bleedings, during which time it obtained no food :—

SERUM.

| | FIRST. | SECOND. |
|---|---|---|
| Water - - - - - - - - - - - - - - | 92·48 | 93.35 |
| Albumen and Salts - - - - - - - - - | 7·52 | 6·65 |
| | 100·000 | 100·00 |

BLOOD.

| | FIRST. | SECOND. |
|---|---|---|
| Water - - - - - - - - - - - - - | 82·48 | 83·47 |
| Albumen and Salts - - - - - - - - | 6·70 | 5·95 |
| Globules - - - - - - - - - - - - - | 10·82 | 10·58 |
| | 100·00 | 100·00 |

The globules have here, it is true, diminished at the second bleeding, but so slightly, that we may attribute this circumstance to the unassimulated chyle which must have been present in the system. In the former case, the animal had been exhausted by previous depletions, and hence possessed no store from which the blood could derive even a small portion of serum, as in the latter instance.—*Records of General Science.—Ibid.*

5. *Researches on the Blood.* By L. Gmelin and F. Tiedemann.—Observers have differed with regard to the presence of carbonic acid in the blood.

Vogel found that under the receiver of an air-pump, lime water was acted on by the disengaged carbonic acid.

Scudamore obtained in the same way, by means of barytes water, a precipitate of carbonate of barytes, equivalent to ⅙ or ½ cubic inch of carbonic acid gas, from six ounces of blood.

Brande procured from one ounce of arterial or venous blood 2 cubic inches of carbonic acid.

On the other hand, Darwin could detect no such acid; and Dr. Davy asserts that it is neither extracted during the spontaneous coagulation of the blood, nor by the air-pump, nor by coagulating the serum by heat, and that serum absorbs carbonic acid in greater quantity than pure water, which would not be the case if it was charged with carbonic acid.

Gmelin and Tiedemann examined with great care the blood of a dog taken from the femoral vein and artery, and placed in different tubes under the receiver of an air-pump. The result was that neither carbonic acid nor any other permanent gas was extricated. To ascertain the accuracy of Davy's statement with respect to the absorbing power of blood being greater than that of water, carbonic acid was allowed to stand over arterial blood for five days, when it was ascertained that 100 measures of blood absorb 120 of carbonic acid. The coagulum appeared blackish-red, and the liquid portion was extremely clear.

Since blood contains no free carbonic acid, it was necessary to ascertain whether any existed in it in a combined state. Vinegar was added to each of the kinds of blood which had been collected, as in the former experiment, with every precaution to ensure accuracy, and was placed under a receiver. A quantity of carbonic acid escaped from both, more abundantly from the venous than the arterial. The arterial blood mixed with vinegar, as well as the venous blood, left over mercury for three weeks, was converted into a blackish brown mass, without being separated into serum and coagulum.

About the same period, without a knowledge of the Heidelberg experiments, Ed. Ch. F. Stromeyer obtained the same results.

How do these facts agree with the present theories of respiration?

Lavoisier conceived that without coming in contact with the respired air, a liquid consisting principally of carbon and hydrogen is absorbed through the pulmonary membranes in the bronchi, and is converted into carbonic acid and water through the oxygen of the inspired air. As this theory does not render it necessary to suppose free carbonic acid in the blood, it is not at variance with the observations of Gmelin and Tiedemann, but the passage of gasses into moist animal membrane, and also the immediate contact between air and blood, cannot well be doubted of. Davy inferred from his results that air passes through the moist coats of the pulmonary vessels, and is taken up by the serum, the oxygen partly forming with the carbon of the cruor carbonic acid, and partly combining with the cruor. When he found that after the inspiration of hydrogen some carbonic acid was expired, though much smaller in quantity than after the inspiration of air, he concluded that venous blood contains some free carbonic acid. According to the observations already given, it appears that the arterial and venous blood contain no free acid, but carbonic acid combined with alkali. And if we suppose acetic acid to be formed in respiration, (for we find it in the blood and in most organic liquids which are exposed to the influence of air in combination with alkalies,) then must the venous blood contain more alkaline carbonate than the arterial, when by the formation of acetic acid a portion of the alkaline carbonates will be converted into acetates.

By means of a barytes solution in an exhausted receiver, they estimated that 10,000 parts of arterial blood contain 8·3 of combined carbonic acid, and 10,000 parts of venous blood 12·3 of acid in the same state, being in the proportion of 2 to 3.

They sum up their views of respiration in a few propositions:—

1. That in the pulmonary cells inspired air is absorbed into the moist membranous vessels, and is thus brought in contact with the blood.

2. The azote of the air is not sensibly absorbed by blood, but almost the whole of it remains in the cells. On the contrary, as oxygen is taken up by the blood abundantly, it flows out of the cells into the vessels in proportion to its absorption, and the mixture of gas remaining in the lungs, must therefore contain more azote and less oxygen than the air.

3. The oxygen taken up by the blood combines partly with carbon and hydrogen, and forms carbonic acid and water, and partly unites with the solid organic compounds contained in the blood. From these proceed acetic or lactic acid, which combines with a portion of carbonate of soda contained in the blood, and drives its carbonate acid into the cells.

4. The acetate of soda loses, in its course through the different secreting organs, its acetic acid, combines again with carbonic acid, after undergoing many decompositions in its passage with the mass of blood through the body, and enters into the lungs on its return as carbonate of soda.

*Is urea contained in the blood after the extirpation of the kidneys?*

The authors directed their attention to this point; which it is well known has been decided in the affirmative by Prevost and Dumas, (*Ann. de Chim.* xxiii.)

On the 14th January, 1832, the right kidney of a dog was removed, and in fourteen days the wound healed.

The left kidney was cut out on the 11th of February, and on the 13th the animal died. The substances taken from its body which were subjected to examination, were—1. The liquid vomited; 2. The blood collected from the great vessels, amounting to two ounces; 3. The bile; 4. The contents of the small intestines. All these substances were dried separately on the water bath, and digested by hot water. The filtered liquid was precipitated by acetate of lead, and the lead removed by carbonate of ammonia. The fluid was evaporated to dryness, and treated with absolute spirits. The residue, after evaporation, was dissolved in a little water, and evaporated with nitric acid in a glass tube. The solution from the blood produced, with a drop of nitric acid, a yellowish, white crystallized precipitate, which was collected on a filter, washed with cold water, and dried. A portion of it heated in a platinum spoon, left a trace of carbon; another part, heated with potash, disengaged no ammonia. A third portion was heated with water and carbonate of barytes. The mixture was digested with absolute spirits, and filtered. This liquid, which was not precipitated by sulphuric acid, gave by spontaneous evaporation, long colorless needles, weighing two milligrames. They were soluble in water and spirits; were dissipated by heat, and precipitated by nitric and tartaric acids; they consisted therefore of urea.

From the vomited matter urea was procured, but in such small quantity as with difficulty to be appreciated. A brownish flocky precipitate was obtained from the bile, not completely resembling urea. No precipitate could be detected in the contents of the small intestines, or from the fæces.

Thus the result of the German chemist's researches is, that urea can be formed without the aid of the kidneys. The French chemists, Vanquelin and Segalis, found no urea in the blood of a dog forty-eight hours after the extirpation of the kidneys; a circumstance which is probably to be ascribed to the short period which elapsed between the operation and the experiment.

*No urea, or sugar of milk, in healthy blood.*

Ten pounds of fresh blood from the cow, evaporated to dryness in the water-bath, were digested with hot water, and again evaporated. The residue was taken up by water, and precipitated by acetate of lead. The filtered liquid was precipitated by carbonate of ammonia, and evaporated to dryness, and the residue digested with absolute spirits. The latter process was repeated, when, by evaporation, a combination of soda, with a fatty acid, remained.

In the solution of the residue, nitric and oxalic acid occasioned no precipitate of urea, but they separated the fat acid (acid of oil.) It should be observed, that by this process they had previously ascertained 2-500 of urea, and 1-100 sugar of milk, to be appreciable. It appears, therefore, that cow's blood contains neither urea nor sugar of milk, or at least in extremely minute quantity.—*Ib. and Poggendorf's Annalen.—Ibid.*

6. *Physiological and Chemical Researches on the blood of the Vena Porta.*—The first No. of the forty-fourth volume of *Rust's Magasin* contains an account of some highly interesting researches by Professor Schultz, respecting the chemical and physiological differences between the blood of the vena porta, and that of the arteries and other veins. The following is a succinct summary of the results, as given in the *Gazette Med. de Paris*, (15th Aug. 1835.)

1st. The blood of the vena porta is in general blacker than other venous blood, although this difference is not always manifest to the sight; it is not reddened by the neutral salts, or exposure to the atmosphere, or by the action of oxygen.

2nd. The blood of the vena porta does not generally coagulate, but when it does, the coagula are less firm than those of the other arteries. In those cases in which it has coagulated, it liquifies entirely or partly at the end of from twelve to twenty-four hours, and produces, as well as that which does not coagulate, a black sediment, upon which is formed clear serum.

3d. The blood of the vena porta contains on an average, when fresh, 5.23 per cent., and when dry, 0.74 per cent. less fibrine than the blood of the arteries and the other veins.

4th. The liquid blood of the vena porta contains generally a little less solid matter (0.18 to 0.3 per cent.) than the arterial blood and the other venous blood.

5th. Its serum contains generally 1.58 less solid matter than the arterial serum, and 0.80 less than that of other venous blood. In the dry state, the first is of an ash-gray, the second yellow, the third greenish-yellow.

6th. The blood of the vena porta contains proportionately more cruor and less albumen; the contrary is the case in the arterial blood; the dry cruor of the vena porta is brownish gray, that of the other veins deep red, that of the arteries bright red.

7th. The blood of the vena porta contains in its solid parts almost twice as much fat as that of the arteries and the other veins. The proportion is as follows:

| | |
|---|---|
| Blood of the vena porta, - - - | 1.66 per cent. |
| Arterial blood, . - - - | 0.92 " |
| Venous blood of the other veins, - - | 0.83 " |

8th. The dry serum of the vena porta contains but 0.27 per cent. more fat than the dry serum of the arteries and the other veins.

9th. The albuminous cruor of the vena porta contains 1.11 per cent. more fat than that of the arterial blood, and 1.21 per cent. more than that of the blood of the other veins.

10th. It is in the fibrin that this difference is the greatest. The dry fibrin of the vena porta contains 10.70 per cent. of fat; that of the arteries 2.34 per cent., so that the difference is 8.36 per cent.

11th. The fat of the blood of the vena porta is blackish brown and unctuous; that of arterial blood and other venous blood white, or yellowish-white and crystalline; that of the white chyle to two-thirds liquid and one-third crystalline.

7. *Case illustrative of the influence of the Ophthalmic Branch of the Fifth Pair of Nerves over the Nutrition of the Eye.*—The morbid phenomena observed in the following case, seem to afford additional confirmation of the opinion entertained by physiologists with regard to the influence exerted by the ophthalmic branch of the fifth nerve over the nutrition of the eye; they nearly correspond with those observed by M. Magendie during his experiments on rabbits, and also with those described by M. Serres, as having occurred in a similar case treated by him.

The principal facts connected with the case now to be noticed, are the following.—William Jolly, three years of age, was admitted into St. Thomas Hospital, under the care of Dr. Burton, on the 29th of January, 1835. The father stated, that the symptoms which his child suffered were preceded, five months previously, by a fit resembling apoplexy; and that upon the child partially regaining his faculties, his right leg and arm were found paralysed. At the time of the patient's admission into St. Thomas's Hospital, these limbs were still almost useless, and the child was unable to stand alone; the sensibility of the paralysed extremities was rather increased than diminished: the head was inclined a little over the left shoulder, in a fixed position, and any attempt to rotate it occasioned much pain. The patient could only rest in a sitting posture, with his body bent forwards, and his head supported on a pillow; and in this constrained attitude he continued five weeks, throughout the day and night, with little change to the date of his death. At the time of his admission, the body was emaciated; the abdomen tumid, and tender on pressure; the complexion pale and unhealthy; the tongue was clean and moist; the pulse at the wrists exceedingly feeble, and the action of the heart over the cardiac region proportionately weak. The child's intellect did not seem impaired; he understood the questions put to him, and his replies were intelligible, although uttered with a little hesitation; the hearing was distinct; the eyes sound, the sight perfect, but the pupils rather dilated.

No other remarkable phenomenon presented itself until about the expiration of a fortnight from his admission; at that date, a swelling and tenderness of both parotid glands, with erysipelas of the left cheek, and a small speck situated about the centre of the left cornea, appeared in quick succession. When the speck first made its appearance, there was little or no redness of the conjunctival vessels, no intolerance of light at any period; the iris was not apparently inflamed, and the movements of the left eye-ball corresponded with those of the right eye. The speck rapidly extended, and the whole surface of the cornea became opaque. The conjunctiva became more vascular, and its surface was moistened with a semi-purulent fluid; but in the course of seven days from its first appearance the secretion was much diminished, and the surface of the conjunctiva had become almost dry. Ulceration through the cornea advanced; and within the laminæ of the inferior half of its disk a purulent fluid had collected, which eventually escaped through the perforation, and projected in the form of a brown-colored conical scab, about one-eighth of an inch from the surface of the cornea. The globe of the eye was now discovered to have entirely lost its perception of external stimuli; no symptoms of uneasiness, and no movements of the eye-lids, followed, when the globe was touched in the first instance by the finger, nor when subsequently touched with lunar caustic. The child could, however, still move the eye-lids, and their movements corresponded, as before remarked, with those of the other eye. The sensitiveness of the left cheek was natural; and from the desire which the child manifested for strong beef-tea, in preference to weak mutton broth, it was inferred at least one of the gustatory nerves was not paralysed; but owing to the distress exhibited by the patient whenever any attempt was made to move him, and the difficulty of making him comprehend the necessary questions, it was not ascertained whether the left nostril and left half of the tongue were paralysed or healthy.

The ulceration continued to progress from the first appearance of the speck about nine or ten days; the eye then burst, and the humors were discharged. A considerable hæmorrhage also followed, and an eruption of petechiæ was then for the first time noticed on the lower extremities. All the symptoms now became rapidly worse. In the course of a day hæmorrhage followed from the bowels, the puerpera ascended over the trunk and upper extremities, and the child sunk exhausted, but only imperfectly comatose, about twenty days after the first appearance of the speck.

Upon examining the contents of the cranium, thirty hours after death, the dura mater was found to be marked with petechiæ over the upper circumference of the cerebrum; and a scrofulus tubercle adhered, or had grown out of the anterior superior surface of that portion of the dura mater which forms the tentorium; in other respects this membrane was healthy. The arachnoid also seemed perfectly free from disease, and the brain firm but not hard. A small quantity of a thin fluid, tinged with blood, was effused between the arachnoid and pia mater, at the upper circumference of the brain. A little fluid was found in both the lateral ventricles, and a loose coagulum of blood in the posterior cornu of the right ventricle. About f. ℥j. of loosely coagulated blood was also extravasated between the convolutions of the left hemisphere of the cerebrum. Many small scrofulous tubercles were discovered in the cerebrum, about the size of peas; two others much larger, about three-quarters of an inch in diameter, were also found in the cerebellum; and one of the same dimensions nearly was situated about the posterior inferior portion of the pons varolii. The circumference of this tubercle was placed within the distance of one line from the apparent origin of the fifth nerve, on the left side of the pons varolii. The brain was carefully examined by Dr. Barker and Dr. Burton, but no other disease could be discovered either in the course of the fifth nerve, on the same side as the tubercle, or in the left cavernous sinis, or in the left orbit. As, however, the phenomena attending the ulcerative process in the above case nearly corresponded with those described by M. Magendie and M. Serres, to have followed the destruction of the healthy functions of the fifth nerve in their cases, we may ascribe the corresponding set of phenomena observed in the eye of W. Jolly to a similar lesion; and in the absence of direct evidence to the contrary, it may be attributed with much probability to the morbid action which occasioned the growth of the tubercle, noticed immediately beneath the apparent origin of the fifth nerve, on the left side of the pons varolii.

The cachectic condition of the fluids and solids of the body was well exemplified by the joint existence of puerpera and scrofulous tubercles to a remarkable extent. Capillary extravasation was observed in the three great cavities of the body, the cranium, chest, and abdomen; and all the principal organs were in a state of scrofulous derangement. The tubercles were as generally distributed in the case of W. Jolly, as they appear to have been in the case described by Mr. Earle, in the third volume of the Medico-Chirurgical Transactions. The abdomen, in particular, presented the characters noticed by Dr. Baillie (page 208, vol. 1, Wardrop's edition,) and the peritoneum and mesentery were thickly studded over with small scrofulous tumors.

The scrofulous habit predominated throughout the family. Of six children, including the patient, William Jolly, five had died about the age of 3 years; the sixth is not likely to thrive; and the mother died, after a protracted illness, in child-bed.

The treatment adopted in the above case was essentially palliative. Temporary alleviation only of the symptoms was derived from the use of remedies; and at the admission of the patient little hope was entertained of his recovery.—*London Medical Gaz. June 6th*, 1835.

8. *Observations on Malignant Pustule.*—*Hufeland's Journal* for October, 1834, contains some highly interesting observations by Dr. Wagner, on the communication of malignant pustule from diseased animals to the human species. Dr. Wagner having heard that on the 22d July, 1834, two persons in the village of Striesa, in Prussian Saxony, had died suddenly—that several others had fallen sick, and that in one farm seven head of cattle, with several pigs, had burst, immediately went there, where he collected the following details respecting the event.

On the herd of cattle returning from pasturage, July 13th, the bull fell suddenly prostrate, and was unable to rise again. At first it was attributed to a simple wound in the back-bone; he was immediately killed, and two peasants, Stack, the gardener, aged 40, and Zeinz, the vine-dresser, aged 30, both robust men, and in excellent health, skined, cut up, and pertook, with several others, of the flesh of the animal as food. Some days afterwards, several other animals belonging to the same farm fell sick in the same manner, shared the same fate, and their flesh was used as food by the same persons. All of them, however, quickly began to complain of uneasy sensations, heaviness in the precordial region, occasional pains in the abdomen, vertigo, etc., especially Stack and Zeinz, who had not only partaken of the flesh of the infected animals, but had handled them, and in so doing, had been wounded in the hand.

Several more animals suddenly burst on the 15th and 18th. On examination, the abdomen was found inflamed, the spleen gangrenous and putrescent, consisting but of a membrane in form of a sac, containing a thick, black liquid; in several places under the hide, especially about the neck, were œdematous tumours. No doubt could now exist, that the malady was the true carbunculous affection. M. Wagner gives it the name of "gangrene of the spleen, (*Milzbrand;*)" from the state of that organ as found in all the infected individuals examined; and to the septic humour which appears to generate the malady, "virus of gangrenous spleen, (*Milzbrandgift.*)

On the 19th, the gardener Stack, though suffering for some days, endeavored to walk a distance of three leagues, which, with the utmost difficulty, he accomplished. After having endeavored to recruit his feeble state with a draught of malt liquor, he made an effort to return, but was seized with vomiting, pains in the abdomen, and fell prostrate on his back. He was carried home; icy coldness of the extremities, thence to the trunk, supervened; diarrhœa of black liquefied blood; convulsive movements of the head and limbs; legs blue and livid; nose sharpened; eyes hollowed; with great suffering in the abdomen, and repeated vomiting; but death relieved him on the 20th.

On the same day, the widow Gaertner, who had eaten of the meat, was affected much in the same manner; a black pustule also had appeared on one of her thighs. She was found dead in her bed on the following day, with a child still sleeping beside her and in perfect health. On the 22d the decomposition of these two bodies was advanced nearly to liquefaction, and, therefore, precluded examination.

Eight other persons who had either come in contact with the infected animals, or had eaten of their flesh, were attacked with the epidemic on the 22d of July, and a ninth on the day following. The general symptoms and sufferings were nearly the same in all. There was dryness of the skin; small, febrile, scarcely perceptible pulse; tongue not loaded; eyes natural, and body red and warm; no delirium. Some experienced a sense of pressure at the epigastrium, which was not, however, increased on drawing a full breath; most of them had a sweetish taste in the mouth; all had inappetence; two only were affected with vomiting; none of them had meteorism and tension of the abdomen. Some suffered anxiety, especially the vinedresser, Zeinz, who, with the exception of a sensation of numbness in the affected thigh, did not complain of any more pain than the others. The anthrax in this individual had no inflammatory circle; it was surrounded by an induration, was sensible to the touch, of an oblong form in the direction of the limb, and extended in depth to the bone. The pustule commenced in a small spot the size of a pin's head, and extended without causing any pain, and had become entirely dry.

The second patient, who had a pustule on the thumb, likewise had no general symptoms; but he had a sensation of burning, with tumefaction and erysipelatous inflammation of the affected hand, which extended to the forearm, although the anthrax was scarcely the size of a ten-cent piece.

With the exception, also, of a young woman, who ate of the infected meat, and in whom a pustule formed on the right forearm, with tumefaction and inflammation extending to the elbow, the other patients had no pustular eruption.

As the malady with most of these persons had already been of some days' duration when M. Wagner arrived, he found no indications requiring vomits. Those who were yet free from anthrax, he administered the most simple remedies; cataplasms of linseed and flour of bran in white wine-vinegar, applied to the precordial region, infusion of fennel, or simply water acidulated with white wine vinegar, to promote and sustain a moderate perspiration; abstinence as far as possible. With those in easy circumstances, and with those also, in whom carbuncles were developed, a more active treatment was necessary. An incision cross-ways was made in the pustules, and, with respect to Zeinz, to the depth of half an inch, cauterised, and the wound sprinkled with strong caustic potass. During nearly the whole operation he was insensible, but was at length painfully conscious of a pricking and burning sensation; the gangrenous scab, which was hard and dry, softened and sunk; a cataplasm of linseed and powdered oak bark in white wine vinegar was applied; small doses of camphor, and a strong decoction of quinquina mixed with a little of Hoffman's anodyne liquid mineral.

All the infected were better the next day (24th except one, an old woman, with a pustule on the thumb, which had been cut and cauterised. Every symptom aggravated; the whole arm swelled and inflamed to the shoulder; the forearm covered with reddish-blue vesicles; face red and burning; intense fever; diarrhœa; extreme prostration; skin dry and hard; sweetish taste in the mouth. Considering this case as altogether hopeless, M. Wagner only applied a cataplasm of new cheese to the wound.

In place of the gangrenous scab on the thigh of Zeinz there was a hollow, half an inch deep, circular and blackish, edge narrow and red; fever gone; appetite, strength and spirits returned. This rapid amendment was preceded by a general and profuse sweat of infectious odour. The wound was sprinkled with quinina and caustic potass, dressed with Baum Arcœus, and covered with the vinegared cataplasm; internal treatment as before.

29th. Sensibility restored to the infected member, but the gangrenous hollow was of twice the depth, and no separation could be discerned between the unhealthy and the sound. It was now dressed with powdered quinquina, balsam of Peru, brown ointment mixt with myrrh and camphor, and again covered with the vinegared cataplasm.

Aug. 1st. The gangrenous sloughs were separated with the bistoury; the wound was now three inches in length, two and a half in circumference, and three quarters in depth, of a clear red, and secreting laudable pus.

5th. Begins to be covered with fleshy pimples and granulations of good appearance.

11th. The whole excavation filled with them; occasional torpor in the feet, followed by a pricking sensation.

14th. Cold tumefaction of the integuments of the limb extending to the abdomen. Application of bags of warm bran; the fleshy pimples have pustulated to such a degree as to require the use of the caustic potash.

18. Cicatrisation of the wound beginning, and will, no doubt, be soon completed.

The old woman, whose case had been considered as utterly hopeless, on the 25th, two days afterwards, rallied considerably, to the great astonishment of M. Wagner. No critical perspiration had here supervened, but the diarrhœa was so excessive that the stools were passed without consciousness. The gangrenous pustule was suppurating the vesicles on the fore-arm had sunk, and the swelling subsided from top to bottom. Same treatment external and internal as in the preceding case. The general state of amendment proceeded, and so rapidly, that on the 29th of July she could walk above a mile. The local affecton was more tardy; the pustule extended all round the thumb to the back of the hand; the scab, though superficial, was hard, horny, dry and black, and when a part of it detached itself a few days afterwards, the suppurating surface beneath emitted a fœtid odour. It was dressed with powdered quinquina, and shortly afterwards bore a favorable appearance, and thus continued to do; and on the 4th Sept. the cure was completed.

On the 6th of August two more patients presented themselves, although eight days had elapsed, during which no case of infection either in man or brute had occurred and every precaution was in use to prevent the spread of the epidemic. The persons affected were two fellow servant women on the same farm, the one 26, the other 50 years of age; and though they had been many times in contact with the infected animals, they had never eaten of their flesh. The elder woman, it appears had, while standing beside the infected old woman above mentioned, been stung by a fly in the inner side of the left arm. The little puncture became painful, swelled, and inflamed, and at length assumed the appearance of a dry and livid pustule. The younger woman could not give so decided an origin to the pustule which formed on the outer side of the right arm; it was surrounded with gangrenous vesicles, swelled and inflamed from the elbow to the shoulder; but it is necessary to state, that the hide of an infected animal had been found in her chamber. Might not this have been the origin? Might not some particles of the flesh or cellular tissue have been still adherent to the skin, and in its recent state have attracted the flies, whose puncture would then most certainly transfuse the virus? Such facts have been before observed, and examples of them are cited by Bertrandi and Monteggia.

Recourse was had to the same internal treatment as in the preceding cases; but as it was too late to incise the carbuncles, and M. Wagner having found that cauterisation after incision extended the gangrene, he only applied cataplasms of new cheese, or of bran and linseed. The internal remedies were also soon changed, and a beverage of curdled milk substituted, mixed with water, or an infusion of fennel.

The young woman's fever was of an inflammatory character; and pains in the chest supervening she was bled in the arm. The state of the pustule rendering the application of caustic potass necessary, it was tried, but soon removed, the pain arising from it being too excessive to be borne.

On the 13th of August, after profuse perspiration, the general symptoms, and also the swelling and inflammation around the pustules, subsided with both the patients.

Three weeks after the first appearance of the epidemic, another and fatal case of infection presented itself.

A young man, 20 years of age, servant on the same farm with the females above mentioned, had not only handled infected animals, but had eaten of their first flesh. Nevertheless, he continued in good health for a fortnight after the appearance of the malady; he was then seized with all its symptoms; a pustule on the forepart of the left arm spread an inch and a half in two days. Profuse sweat ameliorated all the symptoms for a short space, but on the 18th they returned, and with such violence, that on the evening of the same day he breathed his last.

In two villages adjacent to Striesa a few isolated cases of the infection were at the same time observed. Four men in the prime of life, who had not only been in contact with infected animals, but had partaken of the flesh as food, were seized with all the symptoms of the epidemic, but recovered in the course of three weeks.

The following experiment is noticeable. Some fat of an infected animal was melted and thrown to two pigs, two dogs, and two cats; all of them burst while rolling on the grass, which they appeared to do for relief.

From the foregoing facts the author draws these conclusions:—

1st. That the greater or less violence of the malady depends not so much on the presence, number, and size of the pustules, as on the concomitant fever; the pustules being but a product or symptom of the malady, and may be altogether wanting, and the latter still exist.

2d. The carbuncular fever, or gangrenous splenitis, with or without pustules, does not propagate itself by means of miasma in the air, but communicates itself by the ingestion of the flesh of infected animals, by contact with them, and by cutaneous absorption. The animal virus, which appears to be the principle of the malady, is fixed, unalterable, not to be decomposed by any process of cooking, as the foregoing statements prove.

3d. Whether the pustules be excised, cauterised, or not touched at all, the concomitant fever and inflammation proceed in their course, and the duration of the treatment is in no degree abridged; but experience demonstrates that violent measures oppose the curative efforts of nature, and may prolong the malady. If the infection be received internally, excision and causterisation are useless; if on the exterior surface they are only useful in the first state of the pustule, when yet very small; and it is rare that medical assistance is sought. The topical remedies which proved most serviceable to M. Wagner were warm emollient cataplasms, or those of new cheese, powdered quinquina, alone, or mixed with powdered charcoal. It is doubtful whether the administration of quinquina and camphor internally be really useful. But, under whatever treatment, the pustule remains ordinarily from four to six weeks; when the infected die it is frequently during a febrile paroxysm. In a slight attack, a vomit has often proved completely efficacious. Milk drunk in great quantity soon after the ingestion of the infectious substance is of use by provoking sickness and vomiting.

M. Wagner attributes the frequency of carbuncular affections, so observable in summer in the Circle of Schweintz, where he practices, to the great number of pools and ponds of stagnant and filthy water in the vicinity of the Elter, and which evaporate during the great heats.—*Gazette Med. de Paris*, 28 Feb., 1835.—*Ibid.*

9. *Remarks on Cough.* By Robert J. Graves, M.D.—Permit me, gentlemen, to make a few observations here on what is properly termed cough. What is cough?—A sudden and violent expulsion of air from the lungs, produced by forcible contraction of the diaphragm, aided by the abdominal and other expiratory muscles. What is the cause of cough?—Pulmonary irritation. What is the nature of this pulmonary irritation?

Here, gentlemen, is a question which every practitioner should put to himself when called on to treat a case of cough, and what affection is there which so frequently demands our assistance and tasks our ingenuity? How abundant, how varied, are the examples of cough we meet with in our daily practice! How obscure do we not find its nature on many occasions, and how difficult and perplexing its treatment! Where the source of irritation is manifest, where the nature of the disease is simple and easily detected, where, after a proper examination, we can point to some part of the respiratory system, and say, here is the seat of the disease; in such cases, indeed, our course is sufficiently clear, we may proceed with confidence, and practice with success. But how often are we, after weeks and even months of close and painful attention, baffled in our best directed efforts, and forced to admit the humbling conviction that all our remedies are inefficient and useless, and that our character, as well as that of the profession, is likely to suffer in public estimation? How often, too, do we discover with surprise, that the cough which we have been treating for weeks as a pure pulmonary affection, depends not on any primary derangement of the respiratory system itself, but upon the irritation of some distant organ, or upon peculiar conditions of the whole economy?

Before I proceed to inquire into the nature of the various sources of pulmonary irritation producing cough, I wish to remark that the exciting cause, or, in other words, that which immediately precedes and seems to give rise to a tendency to cough, is a sensation of tickling in the mucous membrane of the trachea, close to its bifurcation, and opposite the hollow at the fore part of the neck. It is also a curious fact that this sensation of tickling or itching is peculiar to this situation, being never felt in any other part of the pulmonary mucous system. Whether the disease be seated above, as in case of laryngeal affections, or whether it be below, as in case of disease of the lining membrane or parenchyma of the lung, it is here alone that the tickling sensation is felt. Another circumstance equally remarkable and equally difficult of explanation, is the effect of position in cough. Persons laboring under slight bronchitis, or rather slight inflammation of the trachea, who scarcely cough half a dozen times during the course of the day, will, the moment they lie down at night, be seized with a violent and harassing cough, which may last for several minutes, and sometimes for hours, with little intermission. We can easily understand why empyema or pneumonia of one side of the chest may produce cough in certain positions and not in others, for here we have an obvious physical cause; the accumulated fluid in the pleural cavity in the one case, and the diseased lung, whose specific gravity has been much increased by solidification, in the other, exercise an inconvenient degree of pressure on the sound lung, and hence give rise to irritation and cough, particularly in those positions which favor the operation of these physical causes of irritation. Here, however, the cause of irritation is very obscure. It may (but this I merely offer as an hypothesis) depend on the fluid secreted by the mucous membrane trickling over that part of the trachea where the tickling sensation is felt, the flow of mucus to this part being favored by the recumbent position. That it does not depend on any supposed temporary congestion and irritation of the lung, from the impression made on the skin by cold bed-clothes, I am quite convinced, for I have repeatedly observed it in persons warmly dressed, from merely lying down on a sofa close to the fire. You will, therefore, bear in mind, gentlemen, that although usually, when coughing is induced by any sudden change of position, we may infer that it is connected with some serious lesion of the lungs, or pleura, yet we must not attach too much importance to this symptom in arriving at this conclusion, for cases are occasionally met with in which mere tracheal or bronchial inflammation is attended with the same symptom to a very remarkable degree.

I may observe, *en passant*, that the sensation of tickling, or itching, appears to be almost exclusively confined to the skin. Here it appears to be dependent on slight causes, apparently incapable of producing that modification of nervous sensation, termed pain. In other cases it seems to be connected with the rise and decline of the phenomena which indicate inflammatory action, arising in the first case (where it is generally less observable) from that nervous modification which precedes inflammation, and in the second being connected with some change in the nerves of the part which precedes its return to a healthy condition. It does not appear to affect the mucous tissues, except in a very slight degree, and under peculiar circumstances. It is not observed in the pulmonary mucous tissue, except at that part of the trachea which I have already mentioned, and it does not occur in any part of the intestinal mucous membrane. The only parts connected with the intestinal tube, in which it is felt, are the nose and on the anus, and here it is within the reach of scratching, the ordinary mode of relief. This is a fortunate circumstance, gentlemen, for if any part of your bowels were to itch as your skin sometimes does the annoyance would be quite intolerable. If the presence of lumbrici in the small intestines, instead of producing a troublesome itchiness of the nose, as it often does,—if it produced, I say, a degree of itching equally intense in the mucous membrane of the bowels and stomach, what patient could endure greater torments than a person so afflicted? If ascarides gave rise to as intense a degree of itching with the colon, as they occasion at the verge of the anus, how dreadful would be the suffering thus induced!

Passing over the obvious and well known sources of pulmonary irritation, producing cough, such as bronchitis, pneumonia, &c., the first cause to which I shall direct attention is one of not unfrequent occurrence, and where a mistake in diagnosis may lead to a practice useless to the patient and discreditable to the practitioner. The best mode of illustrating this is by giving a brief detail of a case which I attended with Dr. Shekleton. A young lady residing in the neighborhood of Dorset-street, was attacked with symptoms of violent and alarming bronchitis. The fits of coughing went on for hours with extraordinary intensity; it was dry, extremely loud, hollow, and repeated every five or six seconds, night and day, when she was asleep as well as when she was awake. Its violence was such that it threatened, to use a vulgar but expressive phrase, to tear her chest in pieces; and all her friends wondered how her frame could withstand so constant and so terrible an agitation, and yet she fell not away proportionally in flesh, had no fever, and her chest exhibited nothing beyond the rales usually attendant on dry bronchitis. She was bled, leeched, blistered, and got the tartar-emetic mixture, but without experiencing the least relief. We next tried anti-spasmodics, varying and combining them in every way our ingenuity could suggest, still no change. We next had recourse to every species of narcotics, exhibiting in turn the various preparations of conium, hyoscyamus, opium, and prussic acid, but without the slightest benefit. Foiled in all our attempts we gave up the case in despair, and discontinued our visits. Meeting Dr. Shekelton some time afterwards, I enquired anxiously after our patient, and was surprised to hear she was quite recovered and in the enjoyment of excellent health. *She had been cured all at once by an old woman.* This veteran practitioner, a servant in the family, suggested the exhibition of a large dose of spirits of turpentine, with castor oil, for the purpose of relieving a sudden attack of the colic; two or three hours afterwards the young lady passed a large mass of tape worm, and from that moment every symptom of pulmonary irritation disappeared.

The next kind of cough, in which the cause of pulmonary irritation is often misunderstood, is that which occurs in hysteric females. This kind of cough is one of the most alarming diseases in appearance you can possibly witness; in some it is loud, ringing, incessant, and so intensely violent, that one wonders how the air-cells, or blood-vessels, escape being ruptured. In others it is quite as incessant, occurring every two or three seconds, night and day, but it is not very loud, and, indeed, in some it scarcely amounts to more than a constant teasing hem; in general the pulse is quick, but is the quick pulse of hysteria, not of inflammation or fever. The patient suffers no aggravation of the cough from inspiring deeply, and her countenance exhibits no proof of malaeration of the blood, on the contrary it is blanched and pallid. She complains of variable, or deficient, appetite, headache, cold feet, and irregular or absent catamenia; although the cough continues for weeks, or even months, she does not emaciate like a person in incipient phthisis, although so much disturbed by the cough, and subsisting on so small a quantity of food.

Here the history of a case, a knowledge of the patient's habit, and the use of the stethoscope, are of great value. You will find that the patient is subject to hysteria, that she is generally pale and of a nervous habit; that the attack came on suddenly, and was superinduced by mental emotion, or some cause acting on the nervous system, or else arose gradually as one of the sequelæ of catamenial disturbance, that the heat of skin and state of pulse are by no means proportioned to the violence of the symptoms, and the stethoscope will tell you that the signs of organic derangement of the lung are absent. You will thus be enabled to arrive at an accurate notion of the nature of the disease, and you will save the patient from the useless and often dangerous employment of antiphlogistic means. Bleeding and leeching are, generally speaking, injurious; such cases are best treated by stimulants, anti-spasmodics, and stimulant purgatives, together with change of air, traveling, and the use of chalybeate spa-waters.

The third species of obscure cough to which I shall direct your attention, is one of deep importance for many reasons. It is that species of cough which depends upon pulmonary irritation connected with a venereal taint in the system. That syphilis may attack the pulmonary as well as the cutaneous, osseous, mucous, and other tissues, is not a discovery of modern times; it is a form of the disease long known, and you will find it mentioned by many of the older writers. Since syphilis has been classed by Wilan and others among diseases of the skin, this notion seems to have been either abandoned or forgotten, but, as it strikes me, with very little justice. I entertain a firm conviction, that syphilis may affect the pulmonary as well as it does the cutaneous, or mucous, or osseous tissues, and that a patient, laboring under a venereal taint, may have irritation from this cause set up in the lung as well as in any of those organs in which it is usually manifested. The first person who mentioned this circumstance to me was the late Mr. Hewson, and since that time I have had repeated opportunities of confirming the truth of his opinion. Richter, Alibert, and Paget have well observed, that William and Bateman's classification of diseases of the skin is liable to the paramount objection, that it has no reference to the constitutional origin of cutaneous affections. I have the very same fault to find with modern treatises on diseases of the lungs. Pathologists have indeed inquired most accurately into the numerous morbid changes to which the pulmonary tissue is subject, but they have omitted a no less important part of their task, which is to investigate the states of constitution which originated these changes. The agency, indeed, of scrofula has been inquired into with care, but how little attention has been paid to rheumatism, gout, syphilis, and scurvy, the fruitful sources of numerous diseases of the chest.

By far the most interesting point, connected with this affection, is its diagnosis; on this every thing depends. The great importance attached to the diagnosis arises from the circumstance of this disease presenting symptoms analogous to, and consequently being frequently confounded with, phthisis. A patient comes to consult you for cough; you find him pale, emaciated, and feeble; he sleeps badly, and is feverish at night, and has a tendency to sweat. Here there may be a double source of error. If the disease be mistaken for tubercle, and mercury not given, bad consequences will result; on the other hand, if tubercles be present, the effect of administering mercury will be to precipitate the disease to a fatal issue.

What is the nature of this disease, and how are you to recognise it? Mainly, I answer, by the history of the disease. If the patient's sufferings have commenced at the period of time, after primary sores on the genitals, when secondary symptoms usually make their appearance; if some of his complaints are clearly traceable to this scource; if, along with debility, night-sweats, emaciation, nervous irritability, and broken rest at night, we find cough: and if this group of symptoms have associated themselves with others, evidently syphilitic, such as periostitis, sore throat, and eruption on the skin, then we may, with confidence, refer all to the same origin, and may look upon the patient as laboring under a syphilitic cachexy, affecting the lungs as well as other parts. In forming this diagnosis much caution and care are necessary, and we must not draw our conclusion until we have repeatedly examined the chest by means of ausculation and percussion; if these fail to detect any tangible signs of tubercles, we may then proceed to act upon our decision with greater confidence, and may advise a sufficient but cautious use of mercury. Uuder such circumstances it is most pleasing to observe the speedy improvement in the patient's looks and symptoms; the fever, night-sweats, and watchfulness diminish, he begins to get flesh and strength, and, with the symptoms of lues, the cough and pectoral affection disappaer. I am not prepared to say which of the pulmonary tissues is most usually attacked by the venereal poison, but I believe that it chiefly tends to the bronchial mucous membrane, although, like other animal poisons, *e. g.*, those of measles and scarlatina, it may also occasionally produce pneumonia.

The fourth species of obscure pulmonary irritation, producing cough, is that which is connected with a gouty diathesis. Gout may attack almost every tissue in the body. We may have it in the joints, as you are all well aware of, we may have it in the muscles and muscular aponeuroses, forming what has been termed the rheumatic gout; it occurs frequently in the fibrous tissues, and I have several times observed it in the cellular substance of various parts of the body, forming either diffuse œdema or tumours, which are exceedingly tender to the touch, and which are removed by treatment calculated to relieve the constitutional affection. It may attack the heart, giving rise to true pericarditis, or else to a functional disease with palpitations, a sensation of fluttering and sinking about that organ, and very remarkable intermission of the pulse; or it may affect the stomach, occasioning dangerous spasm, or various dyspeptic symptoms; or it may seize on the intestines, producing irritation, colic, and gouty diarrhœa. I remember a patient, of a confirmed gouty habit, expressing a great deal of surprise at getting an attack of gout in the testicle, for he could not conceive how a disease, which generally affects the joints, could occur in an organ so different in its nature. I replied that the matter could easily be explained; because fibrous tissue, which gout most frequently attacks, enters into the composition of the testicle as well as that of the joints. Indeed the testicle, with reference to the texture of its envelopes and the extent of motion it enjoys, may be said to be provided with a sac-like joint. In like manner, gout very frequently attacks the mucous membrane of the trachea, or bronchial tubes, causing a dry, annoying, and often very obstinate cough. Where this cough comes on along with a fit of inflammation of the joints, its true nature is frequently overlooked, and it is believed to have originated in cold, and to be mere common bronchitis. No matter, gentlemen, what be the cause of inflammation in a gouty habit, no matter what the organ attacked by the inflammation be, it almost invariably assumes the character of true gouty inflammation. If a gouty person sprains a toe, or an ankle, matters, after progressing for a time in the ordinary way, are sure in the end to exhibit a change of character, and the inflamed parts are observed either to grow unexpectedly worse, or to become stationary at a time when a speedy termination of the local affection seemed approaching. This is owing to its being now modified by the constitutional tendency to gout, which localizes itself in the affected part. Precisely the same relations may be often observed between common bronchitis, produced by cold in a gouty habit, and the gouty bronchitis it indirectly produces. Gouty bronchitis often becomes chronic, continuing until it is relieved by a regular fit of gout in the extremities.

The fifth species of pulmonary irritation, in which the source of the disease is more or less obscure, is that which is connected with the scorbutic diathesis. It is important to be aware of this, particularly for those who have charge of the health of the poorer classes, which is almost of more value than that of the rich, for on it their labor and their means of support depend. Among the poor, particularly in the cities where the majority live on salt provisions, the scorbutic diathesis is very prevalent. It manifests itself either in the form of puerpera, or in tendencies to hæmorrhage from the nose, stomach, bowels and bladder. It sometimes attacks the lungs, producing irritation of the bronchial mucous membrane, with cough, and spitting of blood, and occasionally gives rise to pulmonary apoplexy. It is evident that pulmonic cases of this nature, originating in a scorbutic diathesis, produced by confined air, damp lodging, and a salt diet, will require a treatment peculiar to themselves, both during the attack and during convalescence.

The last source of pulmonary irritation, to which I shall direct your attention, is that which proceeds from scrofula. You all know that scrofula has a tendency to attack every tissue in the body, but you may not perhaps be aware, that it may affect those tissues in very different ways, and that scrofulous irritation may manifest itself in various forms, from the most trifling and transitory to the most extensive and permanent. I recollect a case I attended with Dr. Jacob, in which this fact struck me very forcibly. A fine boy of high complexion, precocious intellect, and other marks of the scrofulous diathesis, got an attack of the scrofulous ophthalmia of an intense character, and it required all the skill and ingenuity of Dr. Jacob to save him from blindness. During the period of our attendance, his brother (who was also of a strumous habit,) began to complain of parts of his arm being sometimes a little sore. This circumstance attracted my attention, and on examination I found that several circular diffused swellings, of various sizes, often equalling half a crown in diameter, had successively appeared on different parts of his extremities and body. They evidently depended on inflammation of the subcutaneous cellular tissue, and exhibited a remarkable example of a most transitory local affection, produced by a constitutional cause, for these swellings arose, arrived at their acme, and subsided in the space of ten or twelve hours; they constituted, in truth, the first efforts of the scrofulous diathesis, to localize itself, and, after a few weeks' continuance, they were replaced by distinct and *fixed* scrofulous inflammation of the metatarsal bones.

Here was a very curious and instructive fact. A boy, evidently of a scrofulous diathesis, has circumscribed tumors, which arise, come to maturity of irritation, and subside in the course of a few hours. In some weeks afterwards, scrofulous irritation, in a decided and permanent form, fixes itself in the foot, producing inflammation and ulceration. From this it may be inferred, that scrofula, (for in this case I am firmly convinced these tumors were connected with strumous diathesis) may attack parts not only in its more permanent and destructive forms, but also in a manner so trifling and so transitory, as to subside in a few hours, and leave no trace of its existence. The inferences deducible from this fact are numerous and important, for if scrofula may thus produce an acute and transitory inflammation of the subcutaneous cellular tissue, surely it may occasionally give rise to somewhat similar affections of internal organs, as the bowels, the lungs, etc., and thus may occasion an acute bronchitis, a pneumonia, or an inflammation of the mucous membrane of the intestines, totally independent of the operation of cold, or the usual causes of such affections. It has been too much the custom to refer merely chronic and fixed local inflammations to the agency of constitutional causes. The example before us proves that even the most transitory may have this origin.

Scrofulous irritation may affect either the lining membrane or the parenchyma of the lung, giving rise in the one case to scrofulous bronchitis, in the other to scrofulous pneumonia, two affections which may exist separately or combined, and either of which may prove fatal, with or without the development of tubercles in the lungs. Tubercles have, as I have elsewhere proved, too exclusively engrossed the attention of those who have investigated the pathology of phthisis; they are a very frequent product of the scrofulous diathesis, but the scrofulous bronchitis and scrofulous pneumonia are still more frequent and more important, and do not, as is falsely supposed, depend upon the presence of tubercles in the lungs. The pneumonia, the bronchitis, and the tubercles, where they occur together, are all produced by one common cause,—scrofula. Of this more hereafter.—*Ibid.*—*Ibid.*

10. *Notice of a new mode of preserving Animal Bodies.—Communicated at the Editor's request*, by Henry N. Day.—The following account of an interesting discovery, recently made in Italy, is taken from a pamphlet published in Florence, during the last summer.

The author of the discovery, Sig. Girolama Segato, is already favorably known to the scientific world, as the author and engraver of improved maps of Africa and Morocco. Ardent in the pursuit of science, he traversed the deserts of Northern Africa, and by his researches, corrected and considerably advanced the knowledge of those regions. It was while travelling in these parts, that he received the first hint of this great discovery. In the path of one of those interesting phenomena of the African deserts—a vortex of sand—which his curiosity prompted him to trace, he, one day, discovered a carbonized substance, that upon closer investigation proved to have been originally animal matter, and to have been carbonized by the scorching heat of the sand. He afterwards discovered an entire human carcass, partly black, partly of a sooty hue, about a third less than the ordinary size of man, and all perfectly carbonized. It occurred to him that this accidental process of nature, might be imitated by art, to the perfect preservation of animal substances. To discover *how*, now occupied his whole attention. At the end of some months, devoted to this pursuit, the happy thought flashed upon his mind, which was to lead him to the discovery of the desired secret. Compelled to return to Italy, by a dangerous malady brought on by nearly a week's exposure to an unwholesome atmosphere, in a pyramid of Abu-Sir, which he had entered for the purpose of extending his scientific researches, he was obliged to intermit for a time his favorite pursuit; but after regaining his health, he again gave himself to it with renewed ardor; and after a short time succeeded, to the highest degree of his most sanguine expectations.

The following are some of the results obtained by the discovery.

Entire animal bodies yield as readily to the process, as small portions. They become hard, taking a consistency entirely stony. The skin, muscles, nerves, veins, blood, &c., all undergo this wonderful change; and to effect this, it is not necessary to remove any part of the viscera. The color, forms and general characters of the parts remain the same. Offensive substances lose their smell. Putrefaction is checked at once. What is most wonderful of all, is that if the process be carried only to a given degree, the joints remain perfectly flexible. Skeletons even remain united by their own natural ligaments, which become solid although they retain their pliancy. Moisture and insects never injure them. Their volume diminishes a little; the weight remains almost the same. Hair continues firm in its place, and retains its natural appearance. Birds and fishes lose neither their feathers, membranes, scales, nor colors. The insect preserves its minutest appendage. The eyes in most animals, sparkle as in life, and from their want of motion alone would you suppose vitality extinct.

The following are some of the objects, that have been subjected to the petrifying process, and are now exhibited in the studio of Sig. Segato. One of the first of his experiments, was performed upon a Canary bird, (Fringilla Canaria, Lin.) It is still preserved unaltered, although it is now ten years since the experiment was performed; and it has been submitted to the action of water and of insects. A parrot (Psittacus aestivus, Lin.) retains its original brilliancy of plumage, unimpaired. Eggs of the land turtle, turtles, various tarantulae, a water snake, a toad, various kinds of fish, snails and insects, are in a perfect state of preservation. To these, are added various parts of the human body. A hand of a lady, who died of consumption, preserves the emaciation of the disease and of death. Another of a man is flexible in the different phalangic articulations, and yet unalterable; a foot with the nails perfectly fast, a collection of all the intestines of a child, in their natural colors and forms, with the fecal matters unremoved, the liver of a man who died from intemperance, dark and lustrous like ebony; an entire human brain with its convolutions, of extreme hardness; the skin of a woman's breast, naturally configured; a pate of a girl perfectly flexible, from which the hair hangs in curls; the head of an infant partly destroyed, and discolored by putrefaction. There is also in the cabinet of Sig. Segato, a table constructed as follows. A spheroidal surface of wood contains a parallelogram, composed of two hundred and fourteen pieces, regularly arranged. These to the eye appear like the most beautiful pietre dure that have been produced by nature. Their various colors, polish and splendor, and their surprising hardness would leave no doubt of their stony character. The sharpest file, with difficulty, makes an impression on any of them; some it does not attack at all. These species, are all portions of the human body, hardened by this new process; as the heart, liver, pancreas, spleen, tongue, brain, arteries, etc., etc., all resembling the most highly polished precious marbles. An entire body has not yet been tried, principally on account of the limited resources of Sig. Segato, although the expense would be but about one-tenth of that of embalming by the ordinary process.

Great advantages to science, especially to natural history and human anatomy, are expected to result from this discovery ; and it is even confidently believed that the remains of friends, of men of science and of worth, may be preserved for ages in the exact form and appearance, in which the hand of death found them, with nothing offensive or revolting about them.

As vouchers for the accuracy of the statements contained in the pamphlet, the certificates of many of the distinguished physicians, professors and men of science in Florence, where Sig. Segato resides, are appended. Among them, it is sufficient to mention the names of Sig. Betti, Professor of Physiology; Sig. Zannetti, Professor of Human Anatomy; and Dr. Gazzeri, Professor of Chemistry.—*American Journal of Science and Art.*

11. *Solidification of Turpentine by Magnesia.*—From M. Mialhe's experiments on copabia, and M. Faure's on turpentine, it appears that these oleo-resinous juices are most readily solidified by magnesia. The quantity of the latter, however, that is necessary to form a solid mass varies in using different species of turpentine. In this respect there is a marked difference between the common turpentines, such as those of Bordeaux, and the fine sorts, such as the Venetian, which require a much greater quantity than the former. M. Mouchon found that an ounce of Briançon turpentine mixed with an ounce of hydrocarbonate of magnesia, formed a pilular mass, which was a long time before it hardened even a little, and the pills made from which soon loose their globular form.

An ounce of Bordeau turpentine, with six drachms one scruple of hydrocarbonated magnesia, makes pills which harden very slowly, but eventually become pulverulent.

An ounce of Bordeaux turpentine and eight grains of oxide of magnesium, procured by intense calcination, gives a very soft mass, which does not take on a pilular consistence before thirty-six hours. After a few days it resists, in a slight degree, the impress of the fingers, but is truly fragile for a long time. By augmenting the quantity of magnesia, the hardening of the mass takes place more promptly; but to have pills consistent in a few minutes, and to have a magistral preparation, the proportion of magnesia must be increased to a 50th. Pills thus made are pulverulent in forty-eight hours, are transparent, and have a vitreous fracture.

These facts and experiments of M. Faure, repeated by M. M. Guibourt, Lecanu, and Blandeau, lead to the following practical conclusions:—

1. *Carbonated* magnesia should be preferred and employed in equal proportion to solidify Briançon turpentine.

2. When the turpentine of the *Pinus abies* is to be rendered solid, *calcined* magnesia is preferable.

3. The proportions of both should be smaller according to the length of time since the turpentine was collected.

4. In acting on the turpentine of the Pinus abies, reduced by time to a medium consistence, the solidification is effected in thirty-six hours, by means of a fraction of oxide of magnesium equivalent to about 1-72 of the mass.—*Journal de Chimie Med. July*, 1834.—*Amer. Jour. of the Med. Sciences, February*, 1836.

12. *Diabetes Mellitus cured by Kresote.*—Professor Brendt, having been unsuccessful in his treatment of seven cases of Diabetes mellitus, by the various methods recommended by authors, was induced to try in the eighth, the kresote. The following are the particulars of this case, as given in *Kleinert's Repertorium* for 1835.

The patient was a man, fifty years of age, ill for the last sixteen months; he passed daily seven Berlin quarts of turbid urine, sweet to the taste and smell, and containing a good deal of sugar; the patient was feeble, his appetite very great, and he was tormented by constant thirst; his sleep was disturbed by the frequent necessity of making water, but he had no hectic fever. The treatment was commenced by administering a vomit, which brought away some acid-smelling matters. Rollo's plan of treatment was then employed for some days, and ipecacuanha was given in small doses; this produced no good effect, and instead of the ipecacuanha, eight drops of kreösote were administered in the form of pill every day. The quantity of urine now excreted diminished to three, two and a half, and two quarts per day. It appeared at first to contain a large proportion of alkalies, particularly ammonia, and remained troubled. The dose of the kreosote was gradually increased, and after three weeks Rollo's regimen was abandoned on account of the disgust it excited in the patient. At this time the urine gave the odor of horse's urine, contained less sugar, and exhibited the first traces of urea, though it continued still turbid. Under the common diet and increased doses of kreosote, the urine diminished to two or one quart and a half; it was occasionally clear, gave an acid reaction; the quantity of sugar became daily less, and that of urea greater. After some time the patient's state was evidently improved. He now took twenty-four drops a day, his appetite was good, and his thirst much less, and the urine flowed at from one and a quarter to one quart and three quarters per day. In a few days more it assumed a natural color, contained all the ingredients of normal urine, and ceased to give any trace of sugar. —*Ibid.*—*Ibid.*

13. *On Tic Doloureux.* By William Stokes, M. D.—This is one of the most melancholy and harrassing affections to which the human frame is liable; in some instances, the poor sufferer, after having lived for years in a state of exquisite misery, is at last worn out by the intensity and persistence of his agonies. Such was the fate of the late celebrated but unfortunate Dr. Pemberton. A great deal of light has been thrown on the nature of this affection by the researches of Sir Charles Bell. He seems to have succeeded in establishing several points connected with the nature and seat of this affection, one of the most important of which is that the seat of this disease is in the sentient branches of the fifth pair of nerves, and not, as has been supposed, in the portia dura. He has shown pretty clearly that the portia dura is the nerve which regulates the muscular motions of the face, producing all those modifications of features which we call expression, and also peculiar motions or changes connected with certain states of respiration, in a word, that it is the expressive and respiratory nerve of the face. It is, according to him, never the seat of tic doloureux, and the practice of dividing it for this complaint, is as unscientific as unsuccessful. The division of the portia dura in such cases, not only fails in giving relief, but also entails disgrace on the practitioner, and disfigurement and misery on the patient. *Its effect is paralysis of the muscles of one side of the face, and great distortion*, without the slightest relief. Yet it is a melancholy fact that such operations have been performed. Sir C. Bell's researches, however, have put an end to this malpractice, for he has shown that the fifth nerve is that which supplies the face with sensation, and that it is in its branches the disease is situated. We are then, I think, to look upon this disease as a neurosis situated in the expansions of the facial branches of the fifth pair of nerves. Sir C. Bell relates a very remarkable case, in which the patient had suffered from a series of dreadful attacks, the pain coming on in violent paroxysms. From the accounts given by this patient, and from personal observation, he says, that one could trace with anatomical precision the course and direction of the branches of the fifth nerve, for on the recurrence of an attack of pain, he applied his fingers to his face, and made pressure on the foramina where the different branches of the fifth nerve issue. Having done this, he would press the nerves with all his force, and remain in a fixed posture while the paroxysm continued.

Sir Charles Bell's idea with respect to the cause of this disease, is, that it generally depends on some visceral irritation reflected through the sentient branches of the fifth pair of nerves.

I have told you that this disease is one of the most melancholy affections to which man is subject, it is also one of the most obstinate. A vast number of remedies have been employed or proposed for its treatment, and this affords an illustration of the fact, that the more incurable a disease is, the more extensive is the list of its remedies. A few only are deserving of attention, and these I have already mentioned when speaking of hemicrania, namely, the preparations of arsenic, iron, quinine, and opium. Where these fail after a full trial, Dr. Bright looks upon the case as hopeless. Narcotics in every form and of every description have been employed both externally and internally, but to all these the same remark applies; many of these remedies will give temporary relief, and the physician will flatter himself on the prospect of a favorable termination, but in a short time he is annoyed at finding that the disease has returned and left the patient as bad as ever. Many a time have I seen a poor sufferer excited by hope on receiving temporary alleviation from the use of arsenic or iron, and sinking into despair when he found that his torturing malady returned, and that the remedies which on the first trial gave relief, on a second proved useless. The general principles which should guide you in your treatment are, first, to investigate carefully whether any visceral irritation exists, and remove it as far as possible, taking care at the same time to improve the general state of the patient's health, and the next thing is to allay the sensibility of the nerves of the part, and avoid the exciting causes. In certain cases this disease appears to be connected with an affection of the brain, and this seems to be an explanation of the fact before mentioned, that in some cases, where all specific treatment had completely failed, relief has been obtained by shaving the head and applying ice to the scalp during the paroxysms. I have already mentioned to you a case in which this mode of treatment proved eminently successful. This is a curious fact, and one which being of practical importance, you should hold in memory.—*London Med. and Surg. Journal, Sept.* 27, 1834.—*Ibid.*

14. *Digitalis a specific for Delirium Tremens.*—Dr. Cless, of Wurtemberg states, that he has found digitalis purpurea to be specific in the treatment of delirium tremens. Of 13 cases of this disease, in which he administered the remedy, all but two recovered; these two had a relapse. The digitalis was given in strong infusion, in doses of a spoonful every two hours. After symptoms of narcotism have made their appearance, recovery ensues.—*Med. Correspond. Blatt and Gaz. des Hopitaux.*—*Ibid.*

15. *Pulmonary Hæmorrhage treated with Ergot.* By Dr. Durante de Caserta.—A child, aged 12 years, of sanguine temperament, good constitution, but already in the habit of drinking wine, was attacked with colic. His father, without the advice of a physician, gave him an emetic, and, during his efforts in vomiting, copious hæmoptysis took place. Dr. Durante was called to visit the patient, and found him in the following condition: face flushed; expectoration of floculent blood when coughing; pulse high and febrile. The doctor ordered a copious venesection and iced drinks, acidulated with mineral acids, to arrest the hæmorrhage. These remedies were successful, but on the following day the hæmorrhage was renewed with much greater severity, and it returned at every attack of coughing; alternating with epistaxis. M. Durante then prescribed half a drachm of the powdered ergot, to be divided into six doses, one to be administered every two hours. After this medicine had been taken, the hæmorrhage stopped, and did not return, the pulse abated and in a short time there only remained the symptoms of the catarrhal affection. Another dose of ergot was administered to ensure the cure; then some purgative medicine, cooling drinks, and an opiate at night rendered the cure complete.—*Ibid.*

